# Treatment of keratosis pilaris with laser hair removal as monitored by line-field optical coherence tomography: A proof-of-concept study

**DOI:** 10.1016/j.jdcr.2026.01.023

**Published:** 2026-01-27

**Authors:** Priya Agarwal, Noah Musolff, Ria Sandeep, Madeline Tchack, Babar K. Rao

**Affiliations:** a Department of Dermatology, Rutgers Robert Wood Johnson Medical School, Somerset, New Jersey; b Department of Dermatology, Rao Dermatology, Atlantic Highlands, New Jersey; c Department of Dermatology, Georgetown University School of Medicine, Washington, District of Columbia

**Keywords:** keratosis pilaris, laser hair removal, LC-OCT, line-field optical coherence tomography, treatment monitoring

## Introduction

Keratosis pilaris (KP) is a common skin condition characterized by rough bumps associated with keratin-plugging of hair follicles.^1^ KP is considered a benign condition, with patient complaints stemming from cosmetic appearance and/or pruritis.^1^ It commonly affects the extensor aspects of the upper arms, upper legs, and buttocks.^1^^,^^2^ Though the exact prevalence of KP is unknown, it is estimated to affect 50% to 80% of adolescents and 40% of adults.^3^ Due to its common and benign nature, treatment of KP is often considered unnecessary.^3^ Patients may be advised to use topical keratinolytics such as urea or salicylic acid; however, research into effective treatments is limited.^3^ Recurrence following topical treatment is common, with 1 study finding that 60% of patients experienced recurrent KP lesions within 3 months of stopping.^4^

KP is commonly thought to result from abnormal follicular epithelial keratinization, leading to the formation of an infundibular plug.^2^ Notably, coiled hairs are often present within the lesions.^2^ Therefore, it has recently been hypothesized that KP may not be merely a keratinization disorder but may instead result from a circular hair shaft that ruptures the follicular epithelium, triggering inflammation and disrupting normal follicular keratinization.^5^

Line-field optical coherence tomography (LC-OCT) is a noninvasive imaging modality that visualizes the epidermis and upper dermis in real time.^6^ Functioning as a combination of OCT and reflectance confocal microscopy, LC-OCT has emerged as a powerful tool for the detailed assessment of skin architecture in dermatology.^6^ Therefore, LC-OCT may be a tool that can be used for treatment monitoring in patients with KP, offering greater insight into the etiology of the disease, as well as effective therapeutic options.

## Case report

**Fig 1:**
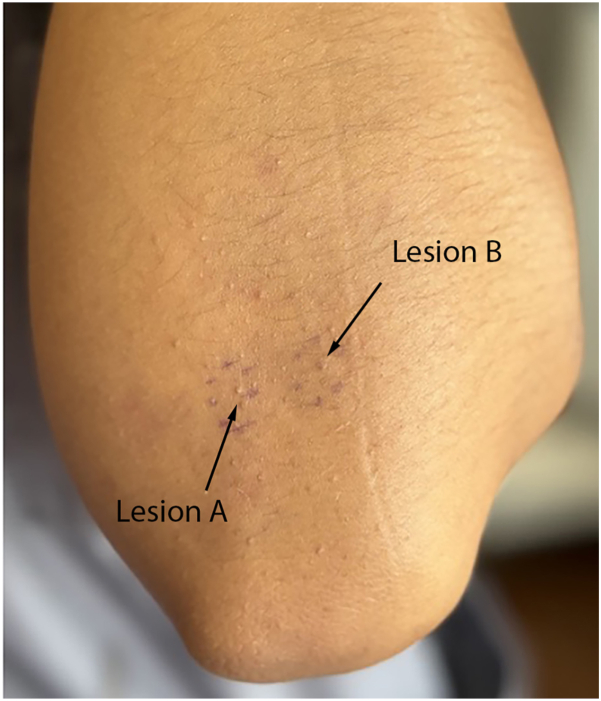
Clinical photograph of the extensor surface of the patient’s left elbow featuring numerous 1-2 mm flesh-colored keratotic papules. The circled lesion on the *left* represents Lesion A (to be treated with triafarotene 0.005% cream), and the circled lesion on the *right* represents Lesion B (to be treated with clinical diode laser).

In this case, a 24-year-old female with Fitzpatrick Type IV skin and no significant past medical history presented to the clinic with numerous cosmetically bothersome “bumps” on her elbows. She reported that the lesions had been present for numerous years and had no improvement with topical exfoliants such as glycolic acid and over-the-counter lactic acid lotion. On examination, numerous 1-2 mm flesh-colored keratotic papules containing hair follicles localized on the extensor surfaces of the elbows, producing a sandpaper-like texture, were noted. Two adjacent KP-affected areas were selected for study (Fig 1). Lesion A (left) was assigned treatment with trifarotene 0.005% cream applied once nightly, and Lesion B (right) was assigned treatment with an 808 nm diode laser delivering 10 J/cm^2^ fluence with a 40 ms pulse width at 4 hz frequency (RAZORLASE SDL-C Diode Laser Therapy System). The treatment sites were positioned side by side to allow for direct visual comparison under identical skin conditions with enough distance to prevent overlap. The patient applied the topical treatment to Lesion A once nightly and recorded applications in a log over the course of the treatment period.

**Fig 2:**
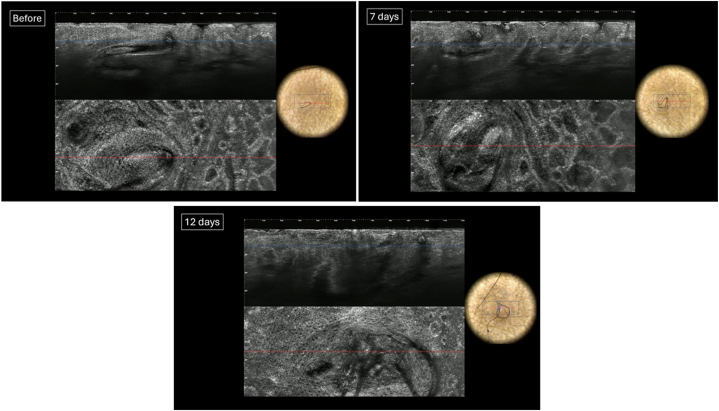
LC-OCT and dermoscopic photographs of KP Lesion A, treated with triafarotene 0.005% cream at T0, T2, and T3, respectively. *LC-OCT*, Line-field optical coherence tomography.

**Fig 3:**
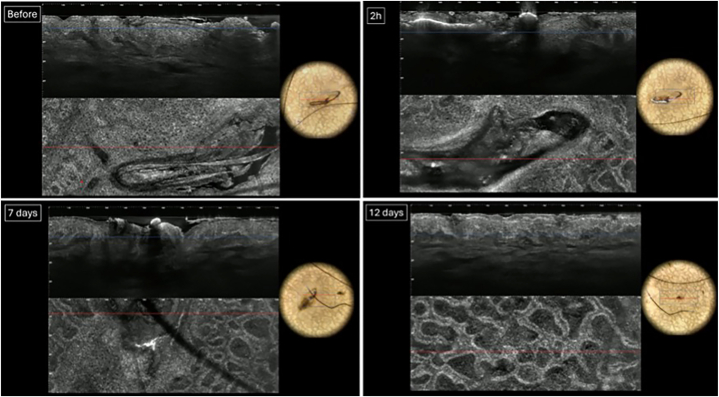
LC-OCT and dermoscopic photographs of KP Lesion B, treated with the clinical diode laser, at T0, T1, T2, and T3, respectively. *LC-OCT*, Line-field optical coherence tomography.

LC-OCT images and clinical photos were taken at baseline (T0), day 7 (T2), and day 12 (T3) for Lesion A (Fig 2). LC-OCT images and clinical photos were taken at baseline (T0), immediately after treatment (T1), day 7 (T2), and day 12 (T3) for Lesion B (Fig 3). LC-OCT, Line-field optical coherence tomography.

**Fig 4:**
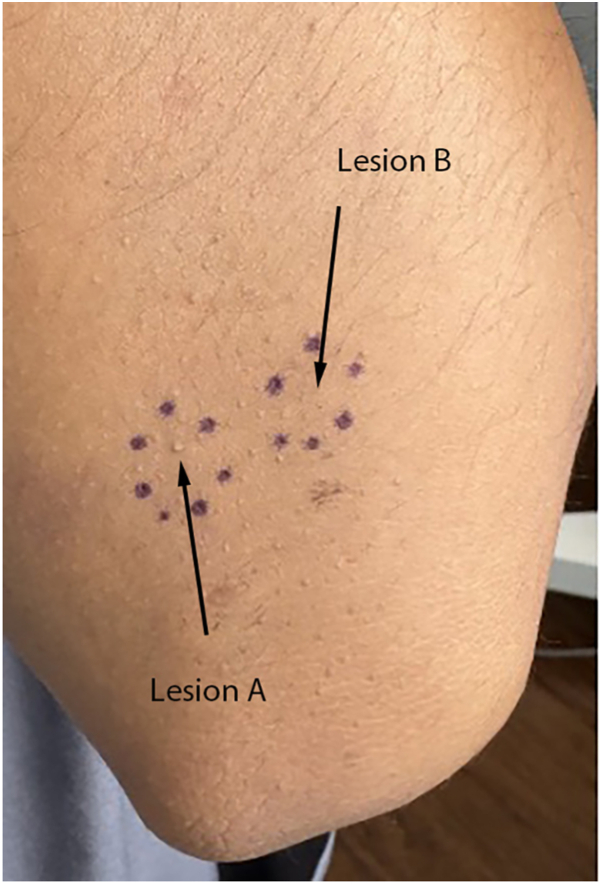
Clinical photograph of the extensor surface of the left elbow on day 12 postlaser treatment. Lesion B (*right*) is noticeably flattened compared to Lesion A (*left*), which appears largely unchanged from day 0.

Pretreatment LC-OCT images of Lesion A revealed hair shafts and follicles within the epidermis and upper dermis. By T2 and T3, the hair shaft is still present with unchanged diameter. There was no visible clinical improvement of the lesion.Pretreatment LC-OCT images of Lesion B revealed hair shafts and follicles within the epidermis and upper dermis, with keratin plugs appearing as heterogeneous hyporeflective material. At T1, laser treatment resulted in necrosis and destruction of the follicles, with LC-OCT showing no discernible hair shaft and hyper-reflective material. By T3, no follicle structures were visible, and there was visible clinical improvement of the lesion (Fig 4).

## Discussion

KP is considered one of the most common dermatologic conditions that affects adolescents and adults.^2^ KP often goes untreated due to its benign nature and, therefore, can result in cosmetic dissatisfaction for patients.^3^ Laser therapy recently been explored for the treatment of KP, and has been found to be effective in numerous studies.^7^, ^8^, ^9^, ^10^ Despite this, a recent survey found that only about 9% of dermatologists reported using laser therapy for KP, instead utilizing topical keratinolytics such as lactic and salicylic acid.^4^

This study used LC-OCT to monitor changes resulting from treatment of KP with triafarotene 0.005% cream versus laser hair removal with a clinical diode laser. In line with a recent study, we considered that if KP results from a circular hair shaft that ruptures the follicular epithelium and triggers inflammation, rather than from abnormal keratinization alone, then treatment with laser hair removal might offer clinical improvement beyond what is typically seen with keratinolytics alone.^5^ We utilized LC-OCT to effectively capture the progression of follicle necrosis and the eventual disappearance of follicle structures after the laser treatment, which correlated with visible clinical improvement not seen in the lesion which was treated topically. This finding offers greater support to the theory that the circular hair shaft is integral to the pathogenesis of KP, and suggests that KP treatment should be targeted to the hair follicle for greater effectiveness. Follicular destruction can be actively monitored via LC-OCT.

This study was intentionally designed as a proof-of-concept evaluation. The findings are limited by the single-subject design and short follow-up period, which restrict broader clinical generalizability and may not have captured the delayed or cumulative effects of topical retinoids or laser treatment. However, the within-patient, lesion-level comparison allowed for a controlled assessment of feasibility and preliminary response. Given the higher cost of energy-based devices, future larger-scale studies comparing diode laser therapy with more affordable topical options over longer periods are warranted to further inform practical and effective treatment strategies for KP.

## Conflicts of interest

None disclosed.
